# Freezing desert soil changes the wind–sand movement pattern

**DOI:** 10.1038/s41598-025-96843-5

**Published:** 2025-04-10

**Authors:** Zhaoen Han, Jinrong Li, Pengcheng Qu, Shaoqi Chai, Yue Liu, Zhenyu Zhao

**Affiliations:** 1 Institute of Desert Control Science and Engineering, Inner Mongolia Agricultural University, Hohhot, 010018 China; 2 Institute of Water Resources and Hydropower Research, Yinshanbeilu Grassland Ecohydrology National Observation and Research Station, 100038 Beijing, China

**Keywords:** Freezing, Wind tunnel test, Soil moisture content, Wind erosion rate, Wind–sand flow structure, Sediment transport rate, Environmental sciences, Solid Earth sciences

## Abstract

In the Ulan Buh Desert, which is located in a seasonally frozen region, a frozen soil layer can appear in the winter after the wind erosion of dry sand from the surface of a mobile sand dune, thus altering the wind–sand transport process. To clarify the wind–sand transport pattern after the emergence of a frozen soil layer, this study used wind tunnel experiments to study the variations in the wind erosion rate and sediment transport pattern of frozen and nonfrozen desert soil with different soil moisture contents (1–5%). The results revealed that the relationships of the wind speed, soil moisture content and wind erosion rate are in line with an exponential function, and the wind erosion rate decreases by 6–52% after the desert soil is frozen. When the soil moisture content of the nonfrozen desert and frozen desert soil is 4% and 3%, respectively, the wind erosion rate of the soil can be reduced by more than 65% compared with that of natural dry sand (soil moisture content of 0.28%), i.e., the wind erosion rate can be effectively reduced. The sediment transport rate of nonfrozen desert soil decreases with increasing height, with an average ratio of approximately 65% for saltation. The sediment transport rate of frozen desert soil first increases but then decreases with increasing height, with an average ratio of approximately 80% for saltation. When sand particles hit the source of frozen desert soil, the interaction between particles and bed surface is dominated by the process of impact and rebound, so that more particles move higher, and some sand particles move from creep to saltation. In summary, freezing has an inhibitory effect on the wind–sand activity of desert soil, and freezing makes it easier for sand to move upwards.

## Introduction

Sediment transport by wind is an important surface process in desert ecosystems^1^and is affected by many factors, including the underlying vegetation, soil, wind speed, topography and human activities. Soil water is one of the important factors affecting wind erosion^2^. The higher the soil moisture content, the stronger the adhesion between the surface sand and water molecules, and the more the sand agglomerates, which increases the threshold wind speed for sand movement and has an inhibitory effect on wind erosion. Moreover, seasonal fluctuations in climate cause corresponding changes in vegetation and soil properties, making soil erosion a time-varying phenomenon rather than a static characteristic^3,4^. In winter, when dunes are covered with snow, have high humidity or are frozen for a long time, the free water in the sand is in a frozen state and closely combines with the sand to form a frozen layer, which affects wind–sand transport to a certain extent^5^. Under the condition of freezing, the sand particles in the surface layer of the soil are more closely bonded. Studies have shown that the wind erosion rate of wet sand is higher than that of frozen sandy soil under the same water content^6,7^. When a critical moisture content is reached, the effect of freezing on wind erosion basically disappears^5^.At present, the critical moisture content measured in the field is about 4%^8^, while the critical moisture content measured in the wind tunnel is about 2%^9^.

By comparing the wind erosion intensity after the soil freeze-thaw cycle with the wind erosion intensity before the cycle, it was found that the soil erodibility increased significantly after the freeze-thaw cycle^10^.This is because during the freeze-thaw process, the large aggregate particles disintegrated and the easily eroded particles increased, resulting in the soil wind erosion intensity after the cycle was higher than 20% before the cycle^11^. By measuring the anti-erodibility of soil samples under different freeze-thaw cycles, it is concluded that there is a critical effect of freeze-thaw cycles on soil anti-erodibility. When the number of freeze-thaw cycles reaches 6 times, the change gradually tends to be stable^12^.

At present, studies on wind–sand motion during freezing periods have focused mainly on Canada^6^, Iceland^13^, Mongolia^14^, the Qinghai-Tibet Plateau in China^15,16^and the black soil region of Northeast China^17^, with a particular focus on changes in soil hydrothermal properties, soil erodibility and sediment transport rates due to freeze–thaw cycles^18^. The Ulan Buh Desert is a seasonal freeze–thaw area adjacent to the Yellow River; the coastal sand activity is active, and the wind–sand flow can enter the Yellow River. In this area, the topography and landforms are unique, the wind–sand transport conditions are complicated, the wind–sand activity of mobile sand dunes is still active in winter^19^, the process of soil freeze–thaw cycles is relatively short, which only occurs within a few days in late autumn and early spring, and the soil is frozen for most of the winter.

The 5–10 cm surface of the moving sand dune is dry sand layer, and the wet sand layer is under the dry sand layer. The soil moisture in the surface layer of the sand dune in winter is higher than that in springr^20^. When the sand dune soil is frozen, the sand bond is coarsened, the content of more difficult and difficult particles increases, and the shear strength and corrosion resistance increase^21^. When the initial water content is different during freezing, the soil wind erosion rate is 40–98% lower than that under non-freezing^22^. Therefore, in the freezing stage of sand dunes in winter, affected by the freezing of soil moisture in sand dunes, the frozen layer is exposed after wind erosion, forming a “bare spots” on the surface, and then the frozen layer is directly affected by wind erosion.

Owing to the complexity of the wind erosion process of frozen soil, effective field monitoring is difficult, and the following questions have not been fully answered. What changes could happen to wind erosion after the frozen layer appears? What is the wind erosion pattern on the surface of the frozen layer? What is the wind–sand flow structure in the frozen layer? In this study, wind erosion and sediment transport in frozen desert soils with different moisture contents were investigated through wind tunnel tests.

## Materials and methods

### Overview of the sampling locations

The soil sample used in this experiment was from the Liuguai Shatou Test Site in Bayannur city, Inner Mongolia, China. This area has a typical mid-temperature continental dry monsoon climate^23^, with dry air and scarce rainfall; the annual average precipitation is 142.7 mm, mainly concentrated from July to October^24^; the average annual evaporation is 2258.8 mm, the annual average temperature is 8.0 °C, the minimum temperature in winter is − 20.47 °C, the number of days below 0 °C is 100, and the average relative humidity in winter is 53.16%. The soil is mainly aeolian sandy soil with a loose structure. The moisture content of the surface soil can reach 0.15–0.35%, and the moisture content of the 0–100 cm underground soil is 1.5–4.6%.

**Fig. 1:**
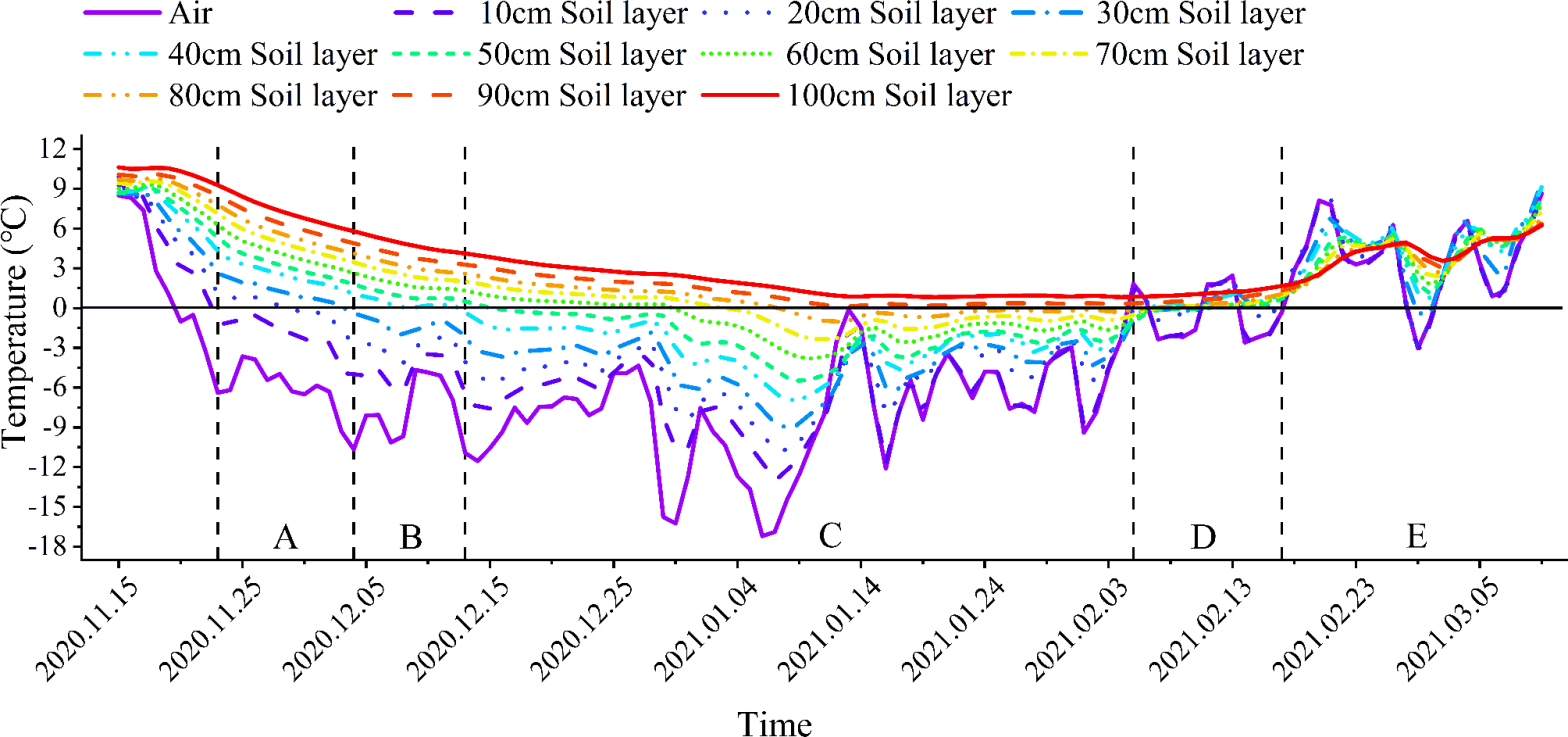
Changes in the air temperature and temperature of each soil layer at the sampling site during the freezing period.

Based on long-term observations, the winter freezing period can be divided into five stages^25^: the initial freezing stage (Fig. 1A), the accelerated freezing stage (Fig. 1B), the stable freezing stage (Fig. 1C), the unstable thawing stage (Fig. 1D), and stable thawing stage (Fig. 1E). The initial freezing stage is from the end of November to the beginning of December, when only the surface soil (0–20 cm) freezes and lasts for approximately 10 days. As the freezing depth increases, in mid-December, the accelerated freezing stage lasts for approximately 10 days and ends when the middle layer (40 cm) of soil freezes. There is a stable freezing stage from mid-December to early February of the following year, which lasts for approximately 55 days. The lowest temperature occurs from the end of December to the beginning of January, and the maximum freezing depth is 81 cm. As the temperature increases, the unstable thawing stage occurs, and the lower and middle soil layers thaw rapidly. The surface 10 cm soil layer experiences day-thawing and night-freezing phenomena, which lasts for approximately two weeks. In mid-to-late February, a stable thawing stage occurs, and the soil layer is completely thawed. The whole cycle lasts approximately 100 days.

In the study area, wind–sand activity is frequent, and surface wind erosion is strong. During the freezing period, westerly winds and northwest winds^26^ dominate, with an annual average wind speed of 3.7 m·s^−1^, a threshold wind speed for sand of 5 m·s^−1^, and a maximum wind speed of 12 m·s^−1^^27^. The multiyear average number of strong wind days is 10–32; for the threshold wind speed for sand, 5–6 m·s^−1^ accounts for 50.26%, 6–10 m·s^−1^ accounts for 47.12%, and > 10 m·s^−1^accounts for less than 3%^28^. Sand-blowing weather occurs year round; the annual average number of sand-blowing days is 75–79, and the number of sandstorm days is 19–22.

## Test methods

### Wind tunnel parameters

A wind tunnel experiment was carried out in the wind tunnel laboratory of the Yinshanbeilu Grassland Eco-Hydrology National Observation and Research Station, Inner Mongolia. The total length of the wind tunnel is 29 m, the diameter of the fan impeller is 1.8 m, the blade angle is 350°, the shaft speed is 720 rpm, and the maximum output power is 185 kW. The entire tunnel is divided into a gas collection section, a power section, a large opening angle section, a stable section, a contraction section, a test section, and a diffusion section. The test section is 12.6 m long × 2.5 m wide × 1.8 m high. The wind tunnel is a DC positive pressure air-blowing wind tunnel with a continuously adjustable wind speed in the range of 2–30 m·s^−1^. Six 2D anemometers and one 3D anemometer with adjustable positions are installed in the test section. The data are transmitted to the computer in the observation room through the wireless network.

**Fig. 2:**
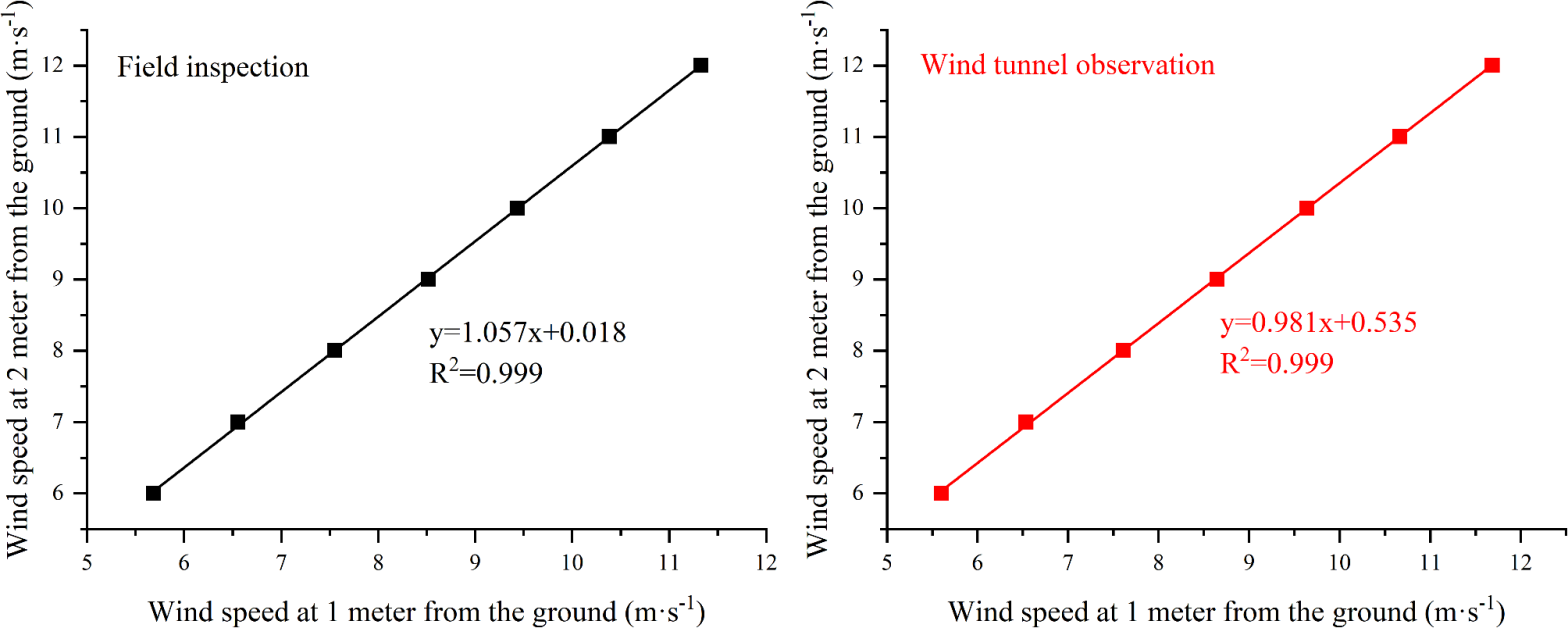
Correspondence between field wind speed and wind tunnel wind speed.

In the early stage, the wind speed was measured on the windward slope of a typical bare dune near the sampling point. The wind speed at 2 m above the ground was used to determine the magnitude of the indicated wind speed, while the Pitot tube measuring the magnitude of the indicated wind speed in the wind tunnel was 147 cm above the ground. Considering the content and purpose of the wind tunnel test, in order to make the measurement results closer to the real value of the field observation, the following processing was done in this experiment : A 5 cm thick sand layer was laid in the wind tunnel test section, and then the wind speed of the test section was measured. The linear relationship between the wind speed at the height of 2 m from the ground in the field and the wind speed at the height of 1.4 m from the ground in the wind tunnel was determined by multiple comparisons. The indicated wind speed at the height of 2 m from the ground in the field can be inverted by the relationship (Fig. 2). In order to make the wind speed profile in the wind tunnel consistent with that in the field, the roughness elements are arranged in the rectification section of the wind tunnel. The wind speed at the test section from 0 to 100 cm above the ground is measured many times, and compared with the wind speed profile at the same height in the field under the same indicated wind speed. The arrangement position of the roughness elements in the rectification section is adjusted by the comparison results until the wind speed profile in the wind tunnel is consistent with that in the field.

### Design of the wind erosion sand tray experiment

To understand the effect of freezing on the wind erosion of desert soil, a wind erosion sand tray test was conducted to understand the dust emission of desert soil after freezing from the perspective of wind erosion. The tests for frozen soil were conducted in early February 2023, and the tests for nonfrozen soil were conducted in late March 2023.

**Fig. 3:**
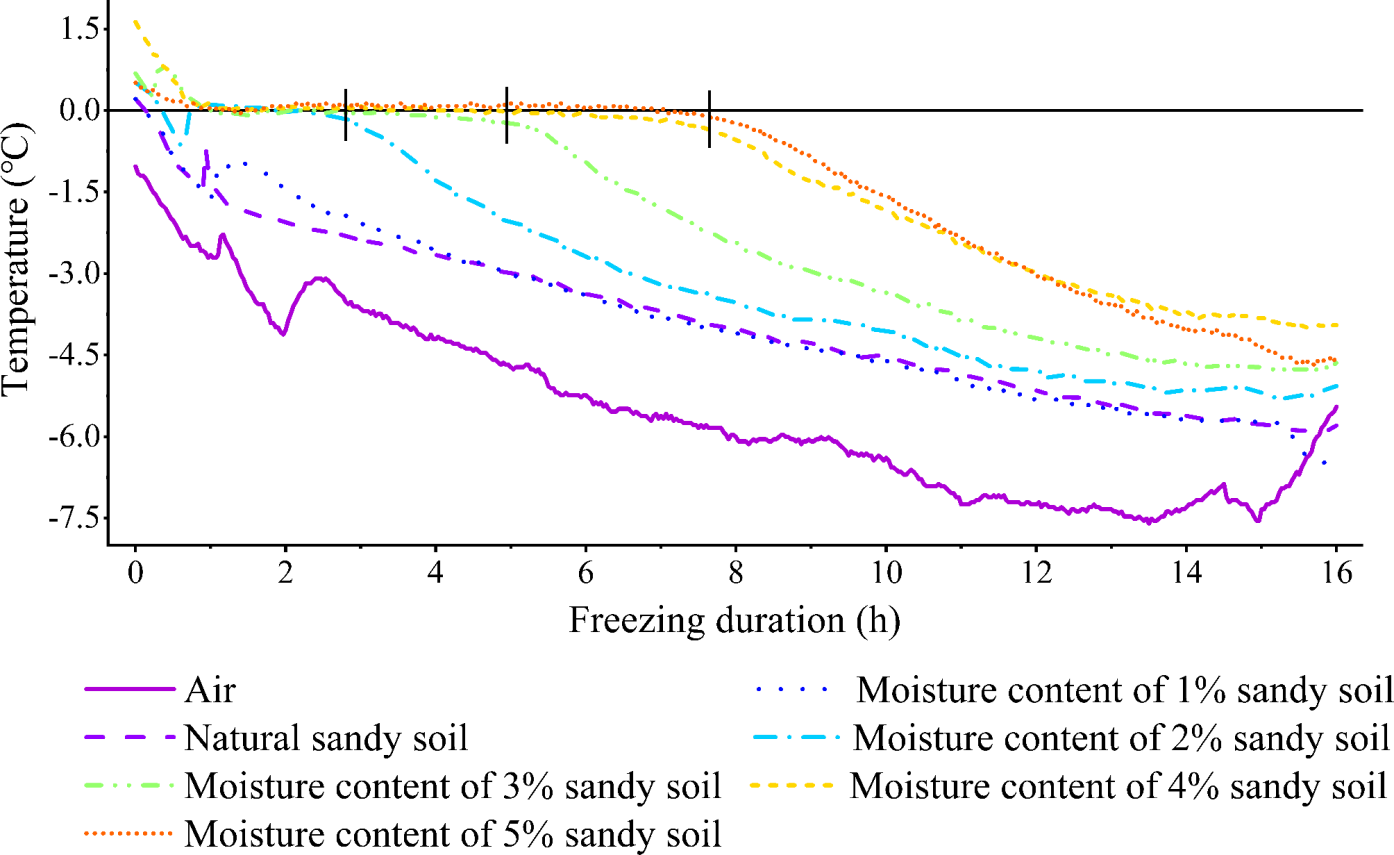
Changes in the freezing temperature of desert soils with different moisture contents.

The previous field investigation and sampling found that the water content of the frozen layer of the moving sand dunes during the freezing period was between 1.4% and 3.7%, and the water content of the dry sand layer was between 0.1% and 0.4%. Therefore, the soil water content was set to 1%, 2%, 3%, 4%, and 5%. The desert soil under natural conditions was used as a control (CK, water content 0.28%). The required amount of water was calculated based on the known amount of dry sand and mixed evenly with the dry sand, and then the samples were put on the sample trays. To ensure the same compactness of each sand tray, the sand trays were lifted to a fixed height then placed naturally on the ground (repeating five times), then the sand tray was wrapped with plastic wrap to avoid water loss. In the freezing test, the natural ambient temperature was used to freeze the soil. A small HOBO thermometer was buried in the sand tray to observe the variation in the soil temperature during the freezing period. The change of freezing temperature of desert soil with different water content gradients was obtained by pre-test (Fig. 3). It was found that when the freezing time was 8 h, the temperature of sand table with water content of 1 − 3% reached below zero, but the temperature of sand table with water content of 4% and 5% was still around zero. When the freezing time was 16 h, the temperature of sand table with water content of 1 − 5% was below zero, and the change tended to be stable. At this time, it was considered that the sand table was in a stable freezing state, so the freezing time was set to 16 h to ensure that all water gradient soils were in a stable freezing state.

**Fig. 4:**
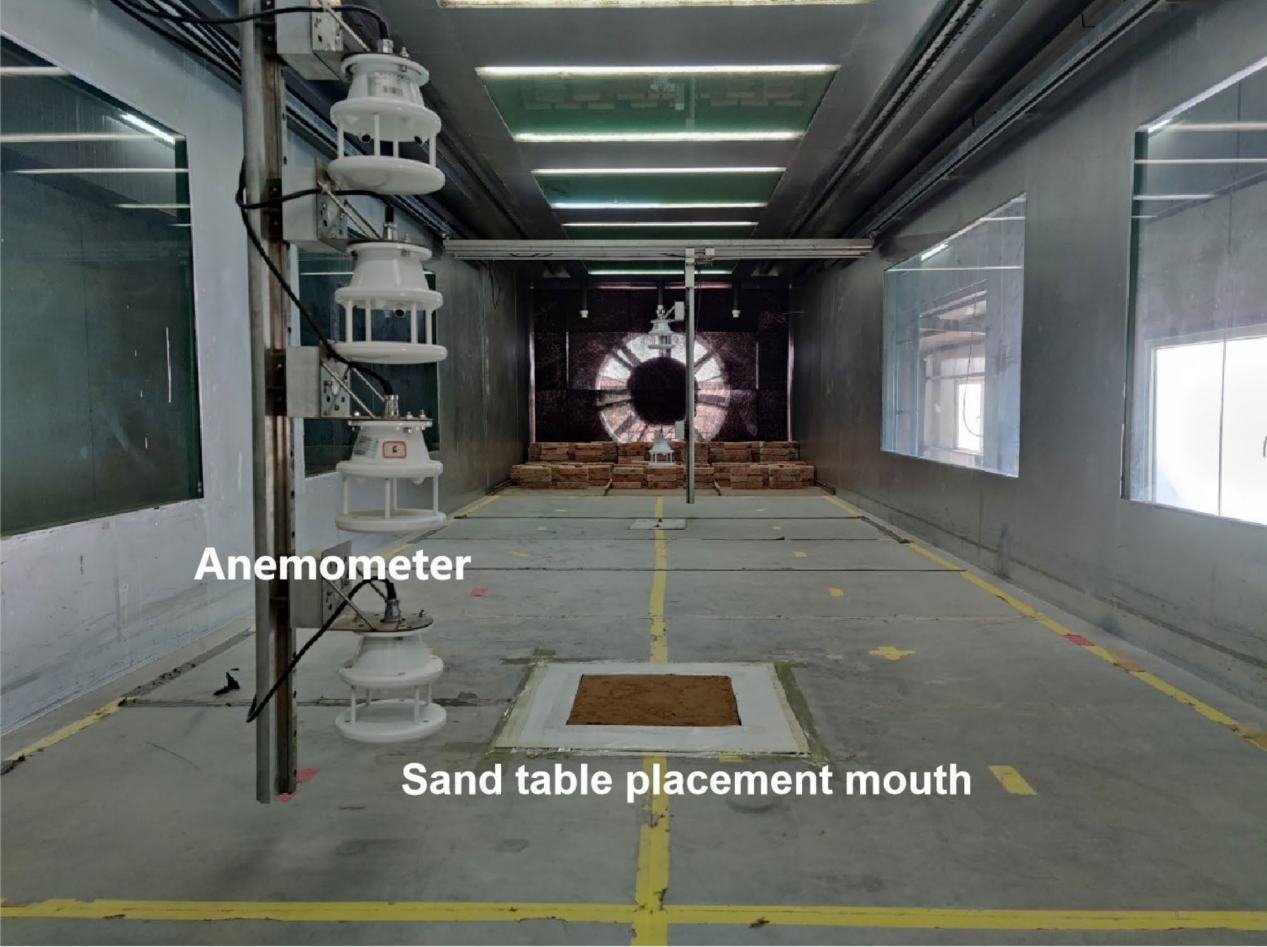
Layout diagram of the wind erosion sand tray.

Sand trays of one moisture content gradient were tested in the wind tunnel laboratory at wind speeds of 5 m·s^−1^ (threshold wind speed), 7 m·s^−1^, 8.5 m·s^−1^ (average wind speed), 10 m·s^−1^, and 12 m·s^−1^ (maximum wind speed). To make the difference in the wind erosion rate significant, the wind erosion time was set to 30 min. During the wind erosion, the sand tray was placed into the mouth of the wind tunnel and was fixed and sealed with iron plates (Fig. 4). The sand tray was weighed before and after wind erosion.

The wind erosion rate was calculated via the following formula:

1 $$Q=\frac{{{M_1} - {M_2}}}{{E \cdot t}}$$

where *Q* is the wind erosion rate, g·m^−2^ ·h^−1^; *M*_1_ and *M*_2_ are the weights of the sand tray before and after wind erosion, respectively, g; *E* is the wind erosion area of the sand sample, m^2^; and *t* is the wind erosion time, h.

### Design of the wind–sand flow observation experiment

**Fig. 5:**
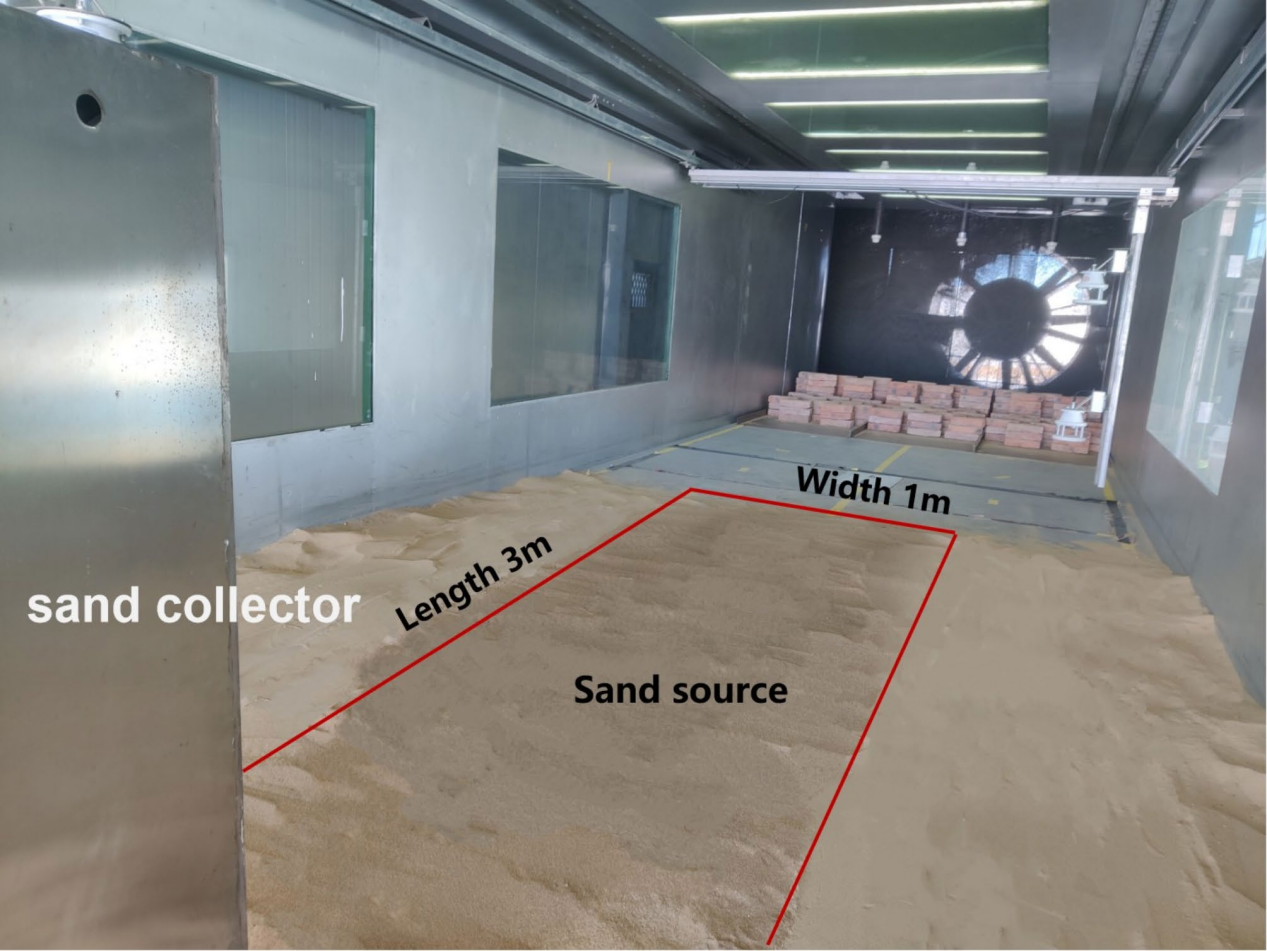
Layout diagram of the wind–sand flow observation experiment.

To further study the effects of freezing on the wind–sand transport environment, an observation experiment on the wind–sand flow of the nonfrozen and frozen desert soil was conducted in a wind tunnel. A 50-layer gradient sand sampler was placed in the test section of the wind tunnel. The size of the sand inlet for each layer of the sand sampler is 2 × 2 cm. The nonfrozen and frozen sand soils with water gradients were paved in front and on both sides of the sand sampler as sand sources (Fig. 5). In order to ensure the smooth test and the full sand source, the sand source was 100 cm wide, 300 cm long, and 5 cm thick. The designed wind speeds were 5 m·s^−1^, 7 m·s^−1^, 8.5 m·s^−1^, 10 m·s^−1^, and 12 m·s^−1^; the wind-erosion time was 30 min, and the sand collected at each layer of the sand sampler was weighed. When the sand material collected by each layer of the weighing sand collector was found, no sand material was collected above 40 cm, so only sand material at the height of 0 ~ 40 cm was collected in this experiment. To more clearly understand the difference in the structure of wind–sand flow between different sand sources, wind–sand flow was analysed layer-by-layer. The wind–sand flow at 0–2 cm close to the bed surface was set as the lower layer of sand flow, the layer of 2–10 cm was the middle layer of sand flow, and the layer of 10–40 cm was the upper layer of the sand flow^29^.

The calculation formula for the sediment transport rate at each layer was as follows:

2 $${{\text{m}}_{\text{f}}}=\frac{{{{\text{m}}_{\text{h}}}}}{{{\text{s}} \cdot {\text{t}}}}$$

where *m*_*f*_ is the sediment transport rate of each layer of the sand sampler, g·cm^−2^ ·h^−1^; *m*_*h*_ is the amount of sand in each layer, g; *s* is the area of the sand inlet of the sand sampler, cm^2^; and *t* is the sand collection time, hour.

## Results and analysis

### Changes in the wind erosion rate

**Fig. 6:**
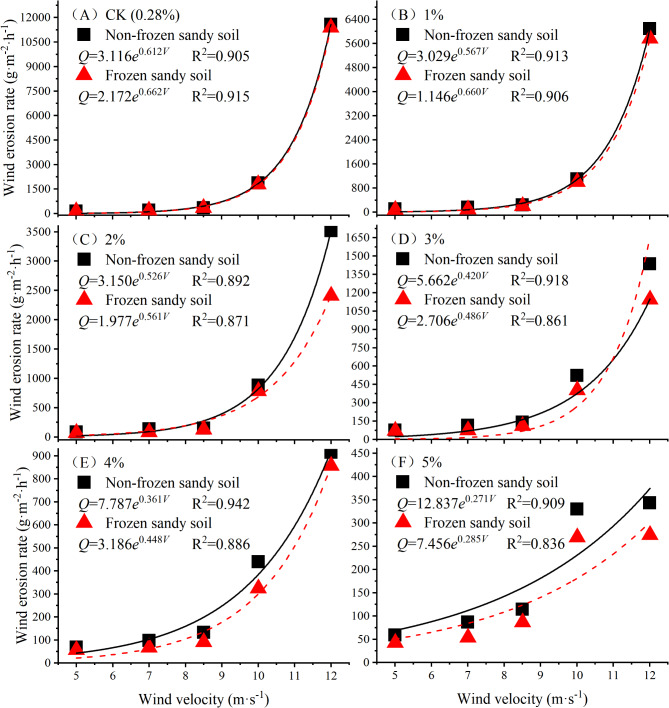
Changes in the wind erosion rate of sandy soil with increasing wind speed.

Under each soil moisture content gradient, the wind erosion rates of nonfrozen and frozen desert soils increase with increasing wind speed (Fig. 6). The wind erosion rate of the nonfrozen desert soil of each moisture content gradient is greater than that of the frozen desert soil. The wind erosion rate of desert soil with a 1% moisture content is 5–39% lower than that of nonfrozen soil, the wind erosion rate of frozen desert soil with 2% and 3% water contents is 10–38% lower than that of nonfrozen soil, the wind erosion rate of frozen desert soil with 4% and 5% moisture contents is 6–36% lower than that of nonfrozen soil, and the reduction in the wind erosion rate tends to decrease with increasing wind speed. In general, under the same moisture content, the wind erosion rate of nonfrozen desert soil is greater than that of frozen desert soil.

The variation in the wind erosion rate *Q* of frozen and nonfrozen desert soil at each moisture content gradient versus the wind speed *V* was fitted by a logarithmic function, a power function and an exponential function. Among the three functions, an exponential function (*Q* = a_1_*e*^*(b1 V*)^) had the best fitting effect. In the function, a_1_ > 0 and b_1_ > 0, where a_1_ reflects the initial characteristics of dust emission and b_1_ is the ratio reflecting the influence of the wind speed on wind erosion. For the nonfrozen condition, the initial values of the exponential functions between the variation in the wind erosion rate and the wind speed of desert soil show an increasing trend, and the ratios of the functions decrease steadily; that is, with increasing moisture content, the degree of skewness of each function decreases, and the degree of influence of the wind speed on the wind erosion rate decreases. The frozen condition is similar to the nonfrozen condition, but the maximum and minimum initial values of the function are lower than those of the nonfrozen condition, and the maximum and minimum ratios of the function are larger than those of the nonfrozen condition.

**Fig. 7:**
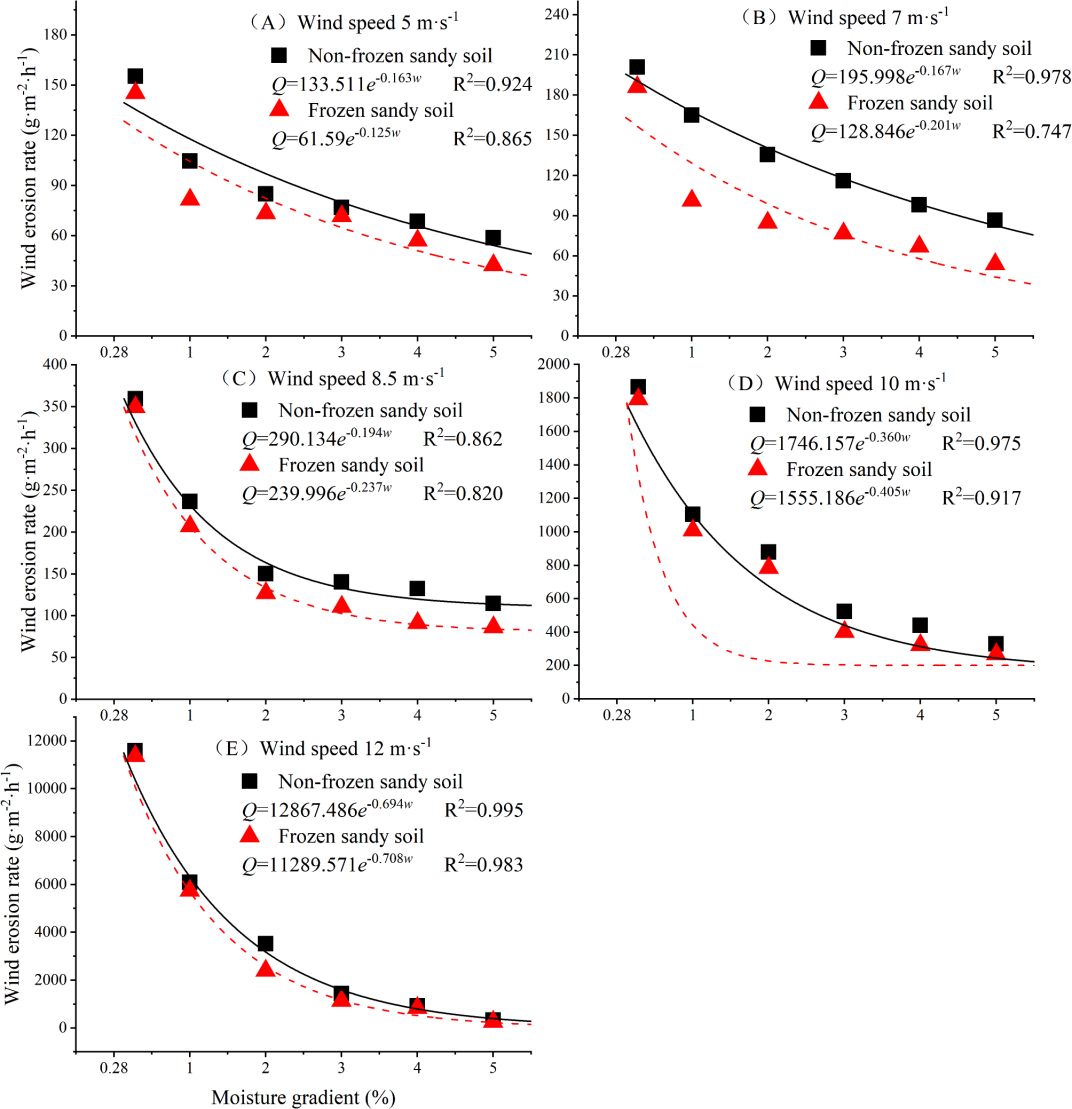
Changes in the wind erosion rate of desert soil with moisture content.

At each wind speed, the wind erosion rates of both nonfrozen and frozen desert soils decrease with increasing moisture content (Fig. 7), and the reduction in wind erosion rate decreases with increasing moisture content. The wind erosion rates of nonfrozen desert soil under different wind speeds are higher than those of frozen desert soil. Under different wind speeds, the wind erosion rate of nonfrozen desert soil with a 1–5% moisture content is 17–97% lower than that of natural dry sand, the wind erosion rate of frozen desert soil with a 1–5% moisture content is 40–98% lower than that of natural dry sand, and the wind erosion rate of frozen desert soil is 6–52% lower than that of nonfrozen desert soil. With increasing wind speed, the reduction in the wind erosion rate of the frozen desert soil is greater than that of the nonfrozen desert soil, indicating that the occurrence of freezing can further inhibit the occurrence of wind erosion.

Under the nonfrozen condition, the wind erosion rate of desert soil with a 1% moisture content decreases by 34% compared with that of natural dry sand, and after freezing, the reduction reaches 44%. The wind erosion rate of nonfrozen desert soil with a 3% moisture content decreases by more than 60% compared with that of natural dry sand, whereas in frozen desert soil, the moisture content is 2%. With a 5% moisture content, the wind erosion rate of nonfrozen desert soil is more than 70% lower than that of natural dry sand, whereas in frozen desert soil, the moisture content is 4%. Therefore, the reduction in the wind erosion rate of frozen desert soil with the same moisture content is greater than that of nonfrozen desert soil. Under the nonfrozen condition, a desert soil moisture content > 3% can effectively inhibit wind erosion, and under the frozen condition, a desert soil moisture content > 2% can effectively reduce the wind erosion rate by 60% or more.

In the Ulan Buh Desert, the multiyear occurrence frequency of winds with a threshold wind speed ≤ 10 m·s^−1^ accounts for more than 97%, and the local surface soil moisture content is between 0.13% and 3% throughout the year; thus, after dry sand in the sample plot is blown away during the freezing period, the exposed frozen layer effectively inhibits the occurrence of wind erosion.

The variation in the wind erosion rate *Q* versus the moisture content *w* for nonfrozen and frozen desert soils under different wind speeds was fitted by different functions. The exponential function was found to have the best fitting effect among the three functions. As the wind speed increases, the initial value of each exponential function for the change in the wind erosion rate of nonfrozen and frozen desert soils increases, and the absolute value of the ratio of each exponential function increases; that is, the initial state of wind erosion increases, and the degree of skewness of each function increases.

#### Variation in the sediment transport rate with height

The variation in the sediment transport rate versus height for nonfrozen and frozen desert soil as sand sources shows that the sediment transport rate at different heights increases with increasing wind speed. When the moisture content is 0.28%, there is no significant difference between the wind–sand flow layers of nonfrozen and frozen desert soil (*P* > 0.1); when the moisture content is 1–5%, there is a significant difference the wind–sand flow layers of nonfrozen and frozen desert soil (*P* < 0.05).

**Fig. 8:**
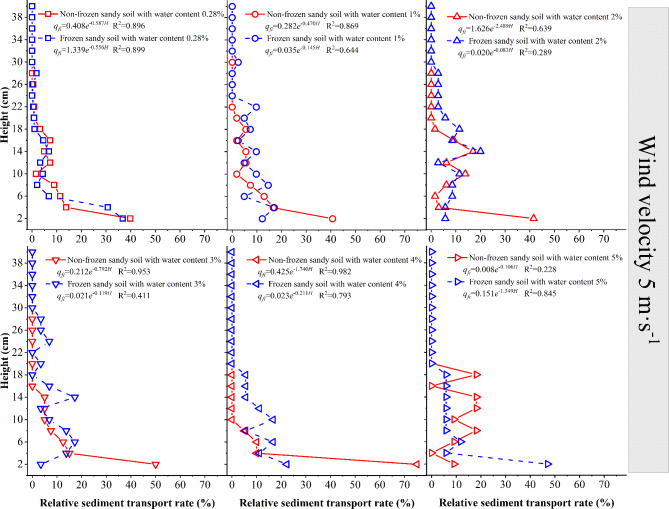
Variation in the sediment transport rate with height at a wind speed of 5 m·s^−1^.

When the wind speed is 5 m·s^−1^ (Fig. 8), the sand source with 0.28% moisture content at the layer of 0–22 cm is in the active sand movement stage. With increasing frozen desert soil moisture content, the sediment transport rate of the lower layer of the frozen desert soil with a moisture content of ≤ 4% is 73–95% lower than that of the nonfrozen desert soil; the sediment transport rate of the lower layer of the frozen desert soil with a moisture content of 5% increases by 87.5% compared with that of the nonfrozen desert soil; the sediment transport rate in the middle layer of the frozen desert soil with a moisture content of ≤ 3% is 6–25% lower than that in the nonfrozen desert soil; the sediment transport rate in the middle layer of the frozen desert soil with a moisture content of > 3% is 20–45% higher than that in the nonfrozen desert soil; the sediment transport rate in the upper layer of the frozen desert soil with water contents of 2% and 5% is 4–34% lower than that in the nonfrozen desert soil; and the sediment transport rate in the upper layer of the frozen desert soil with other water contents is 20–100% higher than that in the nonfrozen desert soil. Therefore, the sediment transport rate in the middle and upper layers of the wind–sand flow in the frozen desert soil is greater than that in the nonfrozen desert soil.

To further understand the variation in the sediment transport rate, a fitting analysis was performed on the variation in the sediment transport rate versus height. Among various functions, such as the dual-parameter exponential function, three-parameter exponential function, dual-parameter power function, and dual-parameter power function, when the wind speed is 5 m·s^−1^, the relationship between the sediment transport rate and the height of each layer of the nonfrozen and frozen desert soil follows a dual-parameter exponential function of *m*_*f*_ =c_1_*e*^*d1H*^. The initial value of c_1_ shows a fluctuating decreasing trend with increasing desert soil moisture content; the absolute value of the ratio d_1_ could reflect the attenuation rate of the sand concentration versus the height; under the nonfrozen condition, the attenuation first increases and then decreases; under the frozen condition, the attenuation first decreases and then increases.

**Fig. 9:**
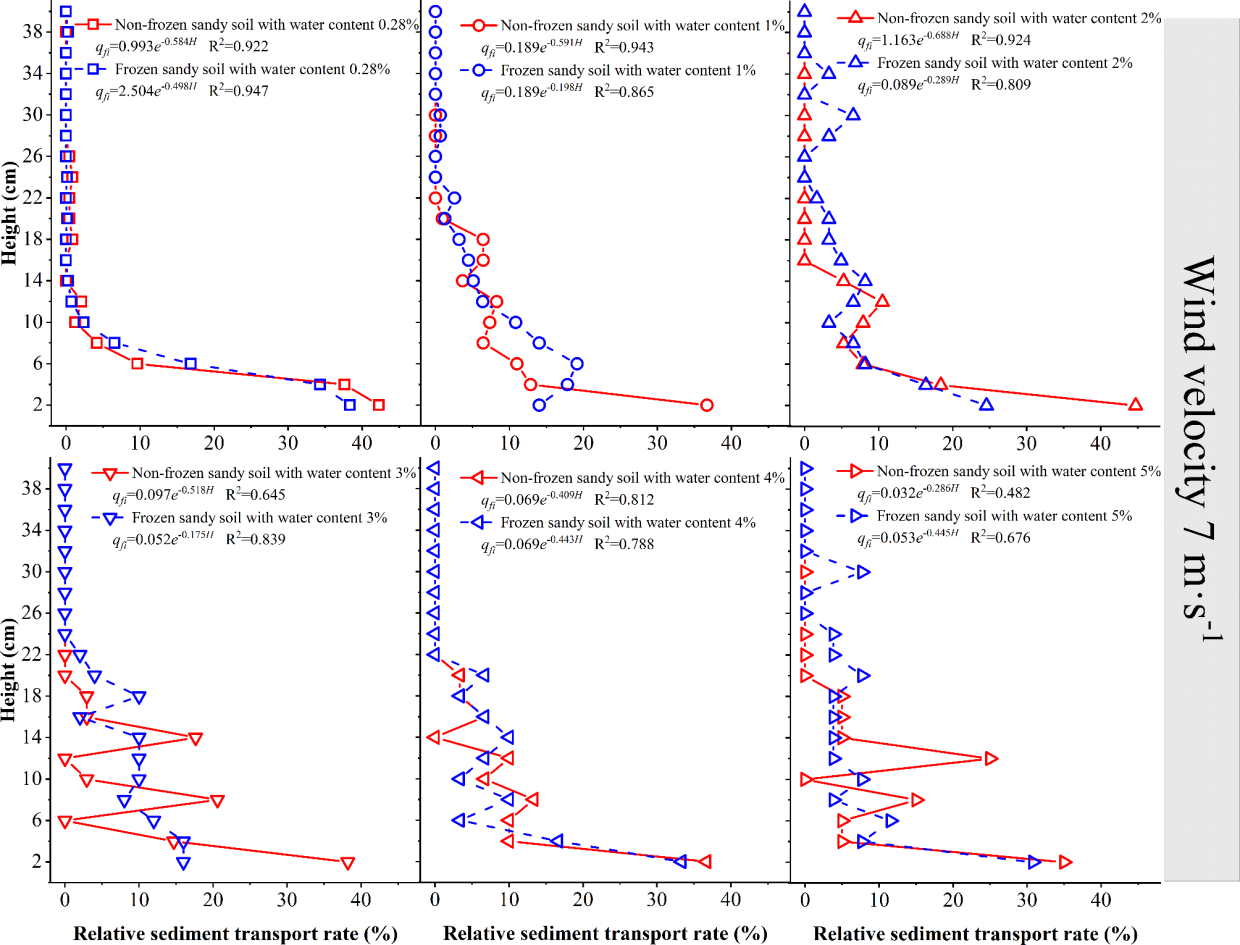
Variation in the sediment transport rate with height at a wind speed of 7 m·s^−1^.

When the wind speed is 7 m·s^−1^ (Fig. 9), the 0–26 cm layer of the sand source with 0.28% moisture content is in the active sand movement stage, and for frozen desert soil with 1–5% moisture content as the sand source, the wind–sand transport activity occurs at the height of 30 cm, 34 cm, 22 cm, 20 cm and 30 cm, respectively. The sediment transport rate of the lower layer of the frozen desert soil with a moisture content ≤ 4% is 9–45% lower than that of the nonfrozen desert soil; the sediment transport rate of the lower layer of the frozen desert soil with a moisture content 5% is 12.5% larger than that of the nonfrozen desert soil; the sediment transport rate of the middle layer of the frozen desert soil with a moisture content 4% is 16.67% lower than that of the nonfrozen desert soil; the sediment transport rate of the middle layer of the frozen desert soil with other moisture contents is 28–58% larger than that of the nonfrozen desert soil; and the sediment transport rate of the upper layer of the frozen desert soil with different moisture contents is 20–74% larger than that of the nonfrozen desert soil.

When the wind speed is 7 m·s^−1^, the relationship between the sediment transport rate of each layer of nonfrozen and frozen desert soil and height is a dual-parameter exponential function. The initial value shows a fluctuating decreasing trend with increasing water content of desert soils; the attenuation increases and then decreases under the nonfrozen condition, whereas it decreases and then increases under the frozen condition.

**Fig. 10:**
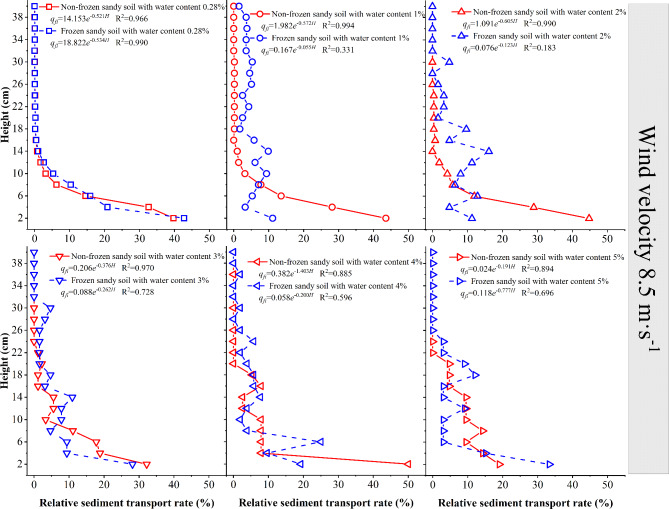
Variation in the sediment transport rate with height at a wind speed of 8.5 m·s^−1^.

When the wind speed is 8.5 m·s^−1^ (Fig. 10), the 0–37 cm layer of the sand source with 0.28% moisture content is in the active sand movement stage, and for frozen desert soil with different moisture contents as the sand source, the wind–sand transport activity occurs at heights of 40 cm, 30 cm, 30 cm, 36 cm, and 24 cm. The sediment transport rate of the lower layer of the frozen desert soil with a moisture content ≤ 4% is 37–94% lower than that of the nonfrozen desert soil; the sediment transport rate of the lower layer of the frozen desert soil with a moisture content 5% is 63.64% larger than that of the nonfrozen desert soil; the sediment transport rate of the middle layer of the frozen desert soil with a moisture content 4% is 42.86% larger than that of the nonfrozen desert soil; the sediment transport rate of the middle layer of the frozen desert soil with other moisture contents is 20–63% lower than that of the nonfrozen desert soil; and the sediment transport rate of the upper layer of the frozen desert soil with different moisture contents is 42–90% larger than that of the nonfrozen desert soil.

When the wind speed is 8.5 m·s^−1^, the relationship between the sediment transport rate of each layer of nonfrozen and frozen desert soil and height is a dual-parameter exponential function. The changes in each parameter are similar to those at 5 m·s^−1^ and 7 m·s^−1^. The initial value shows a fluctuating decreasing trend with increasing water content of desert soils; the attenuation increases and then decreases under the nonfrozen condition, whereas it decreases and then increases under the frozen condition.

**Fig. 11:**
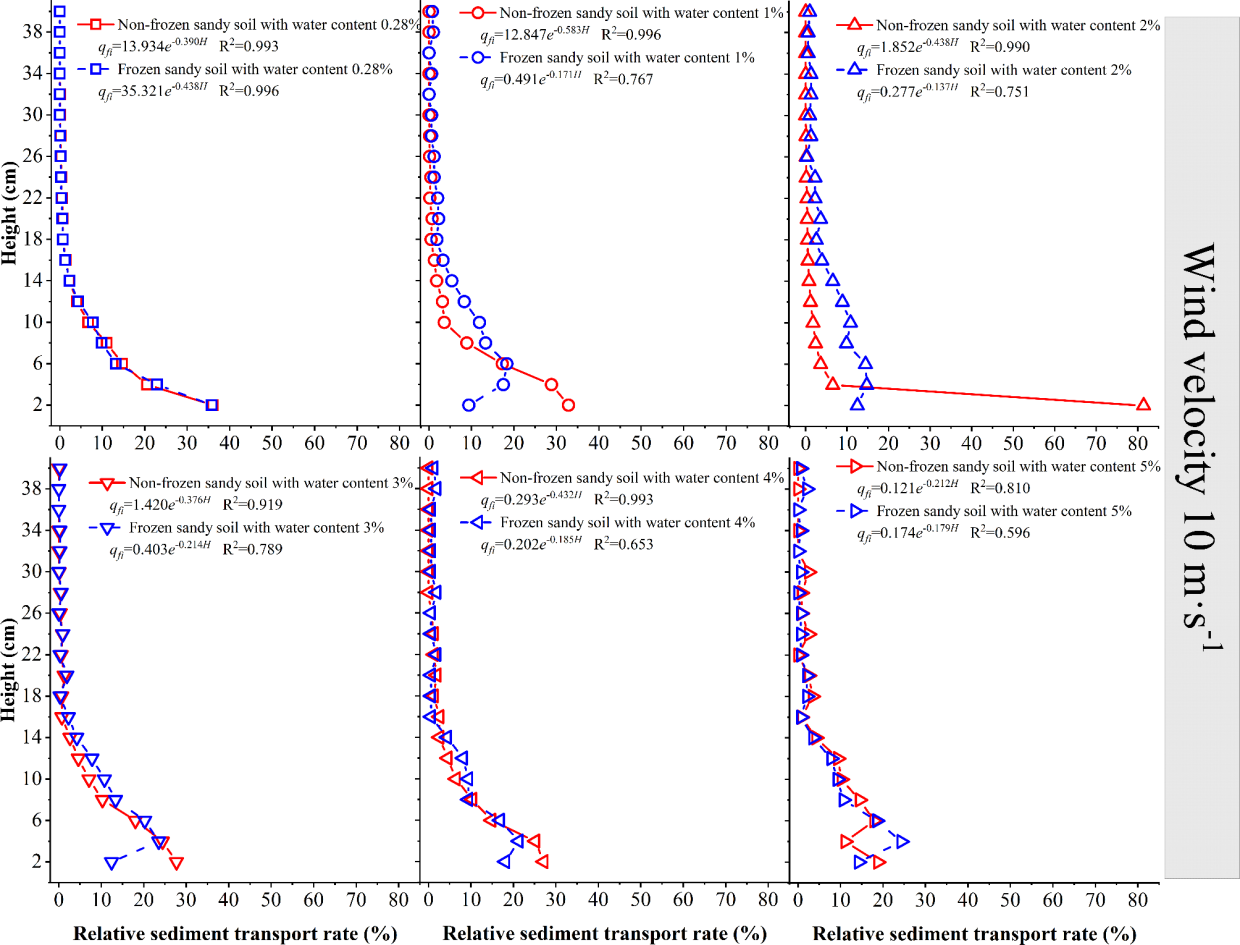
Variation in the sediment transport rate with height at a wind speed of 10 m·s^−1^.

When the wind speed is 10 m·s^−1^ (Fig. 11), the 0–39 cm layer of the sand source with 0.28% moisture content is in the active sand movement stage. For frozen desert soil with a moisture content of 1% as the sand source, the wind–sand transport activity occurs at the height of 38 cm; For frozen desert soil with a moisture content ≥ 2% as the sand source, the wind–sand transport activity occurs at the height of 40 cm. The sediment transport rate of the lower layer of the frozen desert soil with different moisture contents is 9–99% lower than that of the nonfrozen desert soil; the sediment transport rate of the middle and upper layers of the frozen desert soil with a moisture content ≤ 3% is 15–90% lower than that of the nonfrozen desert soil; and the sediment transport rate of the middle and upper layers of the frozen desert soil with a moisture content 4% and 5% is 3–61% larger than that of the nonfrozen desert soil.

When the wind speed is 10 m·s^−1^, the relationship between the sediment transport rate of each layer of nonfrozen and frozen desert soil and height is a dual-parameter exponential function. The changes in each parameter are similar to aforementioned results, with the initial value decreasing as the desert soil moisture content increases. The attenuation first increases but then decreases under the nonfrozen condition, and the attenuation first decreases but then increases under the frozen condition.

**Fig. 12:**
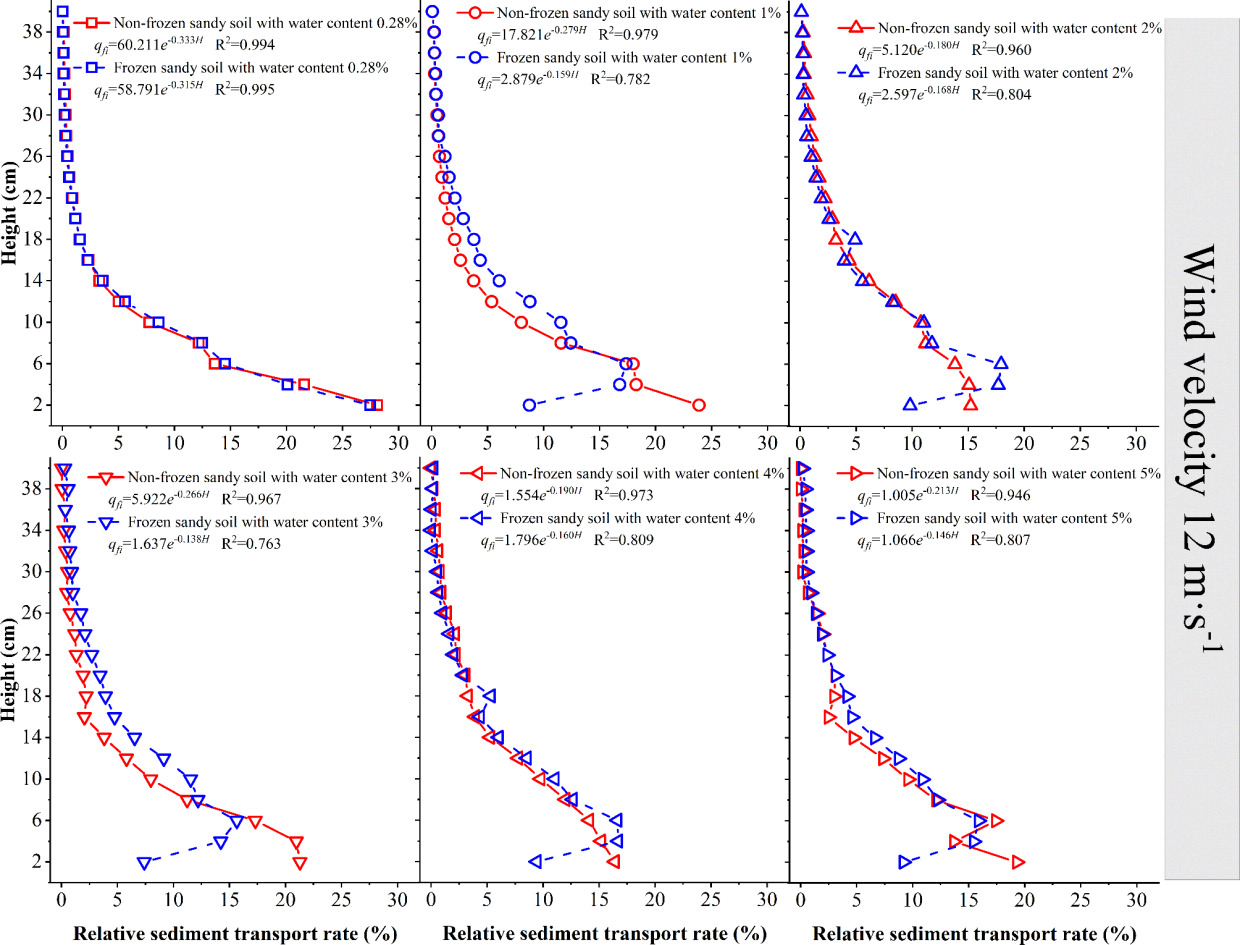
Variation in the sediment transport rate with height at a wind speed of 12 m·s^−1^.

The variations in the sediment transport rate versus height for nonfrozen and frozen desert soils when the wind speed is 12 m·s^−1^ (Fig. 12) are similar to the aforementioned results. When the wind speed is relatively large, the sediment transport rate of each layer of the frozen desert soil with a moisture content < 4% is lower than that of nonfrozen desert soil; however, the sediment transport rate of layers higher than 2 cm of the frozen desert soil with a moisture content ≥ 4% is higher than that of nonfrozen desert soil.

When the wind speed is 12 m·s^−1^, the relationship between the sediment transport rate of each layer of nonfrozen and frozen desert soil and height is a dual-parameter exponential function. The initial value decreases as the desert soil moisture content increases, and the attenuation rate shows a decreasing trend under both nonfrozen and frozen conditions.

In general, the sediment transport rate of nonfrozen sand sources decreases with increasing height. For the wind–sand flows from a sand source with a 0.28% moisture content, the creep (0–2 cm height) accounts for approximately 36%, and the saltation (height of 2–40 cm) accounts for approximately 64%. In the case of the nonfrozen sand source with a moisture content of 1–5%, the creep accounts for approximately 35%, and the saltation accounts for approximately 65%. The sediment transport rate of the frozen sand source first increases but then decreases as the height increases, which is named as the “elephant trunk effect”. In the sand flow from the frozen desert soil, the creep accounts for approximately 20%, and the saltation accounts for approximately 80%, which is greater than the proportion of saltation of the nonfrozen sand source. When the wind speed is 10 m·s^−1^, the maximum sediment transport rate of the frozen desert soil with a moisture content of 1–5% occurs in the 2–4 cm layer; when the wind speed is 12 m·s^−1^, the maximum sediment transport rate of the frozen desert soil with a moisture content of 1–5% occurs in the 4–6 cm layer, indicating that the sand gains more kinetic energy in the collision with the frozen layer, the height of saltating sand grains is larger, and more sand moves to higher layers.

#### Wind–sand transport model

Correlation analysis was performed on the wind erosion rate, wind speed, and moisture content of desert soil under nonfrozen and frozen conditions. The results revealed that there is a significant correlation between the wind erosion rate, wind speed, and desert soil moisture content (*P* < 0.01). To better fit the variation in the wind erosion rate for nonfrozen and frozen desert soils with different frozen moisture contents under different wind speeds, the nonlinear surface fitting with an exponential function, a logarithmic function and a power function on the relationships among the wind speed *V*, desert soil moisture content *w* and wind erosion rate *Q* was performed, and the functions reaching the convergence and with the highest R^2^ were selected. The function for the nonfrozen condition is *Q* = 2.726e^(0.676*V* −0.610*w*)^ (R^2^ = 0.919), and the function for the frozen condition is *Q* = 0.367e^(0.849*V* −0.67*7w*)^ (R^2^ = 0.994).

The relationships among the total sediment transport, wind speed and moisture content in desert soil under nonfrozen and frozen conditions were similar to those of the wind erosion rate. Under the nonfrozen condition, there is an extremely significant correlation (*P* < 0.01) between total sediment transport and wind speed and a significant correlation (*P* < 0.05) between total sediment transport and moisture content. Under the frozen condition, there is a significant correlation (*P* < 0.05) between total sediment transport, wind speed, and soil moisture content. Nonlinear surface fitting was performed on the relationships among the wind speed *V*, desert soil moisture content *w*, and total sediment transport *q*, and the functions reaching the convergence and with the highest R^2^ were selected. Under the condition with nonfrozen desert soil as the sand source, the function is *q* = *e* ^(0.545*V* −0.565*w* −0.403)^ (R^2^ = 0.992). Under the condition with the frozen desert soil as a sand source, the function is *q* = *e* ^(0.648*V* −0.665*w* −1.412)^ (R^2^ = 0.956).

Multiple stepwise regression analysis revealed that wind speed and moisture content can explain 63.39% and 36.31% of the wind erosion rate of nonfrozen desert soils, and 59.69% and 36.40% of the wind erosion rate of frozen desert soils, respectively. The wind speed is the most important factor affecting wind–sand transport, and as the wind speed increases, wind–sand activity becomes more intense; followed by moisture content, and the increase of moisture content has an inhibitory effect on the wind–sand activities.

## Discussion

Desert soil transport is affected by many factors, such as the underlying vegetation, soil, wind speed, topography, and human activities^30^. Owing to different seasons, the vegetation and soil properties in different areas change to varying degrees. There are many studies on the inhibitory effect of the soil moisture content on wind erosion, but there are controversies on the optimal range of the soil moisture content for wind erosion resistance. Cornelis et al. showed that the soil wind erosion modulus decreased with increasing soil moisture content, and winter irrigation can effectively improve the wind erosion resistance of farmland slopes^31^. Chen et al. revealed that when the wind speed was 7.5 m/s and the soil moisture content exceeded 4%, the rate of reduction in the wind erosion rate decreased^32^. Bisal et al. conducted wind tunnel experiments and revealed that when the wind speed was 7.6 m/s, a moisture content of 4% was the threshold for wind erosion resistance; as the wind speed increased to 12.5 m/s, this threshold increased to a moisture content of 6.6%^33^. With a wind speed of 8.5 m/s, the threshold moisture content determined in this study is 3%, which is lower than that found by Bisal et al. At the same time, under the wind speed of 8.5 m/s, the threshold moisture content of frozen desert soil is further reduced to 2%. Additionally, we found that the threshold moisture content increases with increasing wind speed. Under the same wind speed, the threshold moisture content of frozen desert soil is lower than that of nonfrozen desert soil. This phenomenon may be related to the weak binding between water molecules and sand grains in nonfrozen desert soil, resulting in smaller aggregate particles and, as a result, higher threshold wind speeds than those of dry sand. In frozen desert soil, water molecules condense into ice, which significantly increases the binding force with sand grains to form new aggregates of higher mass, increasing the threshold wind speeds and decreasing the likelihood of wind erosion. Therefore, freezing can effectively suppress wind erosion.

In addition, A number of studies have shown that with global warming, surface wind-sand activity may be further intensified^34^. On the one hand, the wind speed tends to increase with increasing air temperature; on the other hand, the temperature during the freezing period of the seasonally frozen area increases, and the soil moisture content decreases, which increases the susceptibility of the sand surface to wind erosion.

To better describe the changes in the wind erosion rate and sediment transport rate, the current mainstream approach is to construct a functional model of the change in the wind erosion rate, and the functional model varies with the underlying surface. The function of the wind erosion rate versus the wind speed with the farmland soil as the underlying surface is an exponential function^35,36^, and the function with the desert soil as the underlying surface is a power function^37–39^. This study revealed that under nonfrozen and frozen conditions, the relationships of wind erosion rate of desert soil with the wind speed and moisture content follows a single-factor variation model, and the dual-factor variation models can be effectively described by an exponential function.

At each moisture content, freezing changes the characteristics of wind–sand transport, resulting in differences in surface sediment transport. Owing to the difference in wind speed and the underlying surface, the function relationship between the sediment transport rate and the height is controversial. In desert areas, the wind–sand flow within 1 m of the near-surface layer decreases with height according to an exponential function^40,41^or a power function^42,43^, and the results of wind tunnel tests tend to be expressed by an exponential function^44,45^. This study revealed that the vertical distribution of the sediment transport rate of desert soil before and after freezing is exponential.

Field studies and wind tunnel tests both revealed that the “elephant trunk effect” occurs when wind–sand flow passes over the compacted bed surface of wet sand^46,47^. In the wind tunnel tests, when the wind–sand flow passes through the dry sand layer, the sediment transport rate decreases with increasing height, with an average proportion of 64% for saltation; when the wind–sand flow passes through the wet sand layer, the sediment transport rate decrease with increasing height, with an average proportion of 65% for saltation, and the “elephant trunk effect” does not appear. When the wet sand is frozen, the wind–sand flow passes through the frozen layer, and the sediment transport rate first increases and then decreases with height, showing the elephant trunk effect, and the average proportion for saltation is approximately 80%, which is 15% greater than that of the nonfrozen layer.

It can be seen that freezing will not only inhibit the generation of wind erosion, but also lead to the change of wind-sand flow. When the frozen sand surface releases sand particles, sand particles are more likely to jump on the frozen surface. The interaction between particles and bed surface is mainly dominated by the impact and rebound process^46^, so that more particles move to higher places, and the movement mode tends to shift from creep to jump.

## Conclusions

In this study, the soil of mobile sand dunes in the Ulan Buh Desert was taken as the study subject, and the effects of freezing on desert soil were investigated through wind tunnel tests. The study revealed that the wind erosion rates of both nonfrozen and frozen desert soils increase with increasing wind speed, with the wind erosion rate of frozen desert soils being 6–52% lower than that of nonfrozen desert soils. We further found that the wind speed and moisture content are the main factors affecting the wind erosion rate of desert soil, with explanatory rates ranging from 51 to 63% and 36–38%, respectively. Under the same wind speed, the threshold moisture content of nonfrozen desert soil is greater than that of frozen desert soil, and it increases with increasing wind speed. Specifically, desert soil with a moisture content > 5% can effectively inhibit wind erosion under the nonfrozen conditions, whereas under the frozen condition, desert soil with a moisture content > 3% can effectively inhibit wind erosion. In addition, the sediment transport rates of nonfrozen and frozen desert soils increase with increasing wind speed. The sediment transport rate of nonfrozen desert soil decreases with increasing height, with an average ratio of approximately 65% for saltation. The sediment transport rate of frozen desert soil first increases but then decreases with increasing height, with an average ratio of approximately 80% for saltation. Therefore, after sand hits the frozen desert soil, the movement mode of the sand tends to shift from creep to saltation. These study results provide an important basis for an in-depth understanding of the interaction between the frozen layer and wind erosion in the desert environment and help to better assess the wind–sand environment in the desert in the seasonally frozen region.

## Data Availability

The data that support the findings of this study are available from the corresponding author upon reasonable request.
